# A network meta-analysis protocol of conservative interventions for urinary incontinence in postpartum women

**DOI:** 10.1097/MD.0000000000021772

**Published:** 2020-08-14

**Authors:** Yang Wang, Hui Li, Jun Wang, Qinghong Hao, Yang Tu, Yalin Chen, Mimi Qiu, Wei Peng, Yunlu Liu, Tianmin Zhu

**Affiliations:** a School of Rehabilitation and Health Preservation, Chengdu University of Traditional Chinese Medicine; b Department of Rehabilitation, Shuangliu Maternal and Child Health Care Hospital; c School of Medicine, Chengdu University; d School of Acupuncture and Tuina, Chengdu University of Traditional Chinese Medicine; e Institute of Laboratory Animal Sciences; f Department of Pharmacy, Sichuan Academy of Medical Sciences & Sichuan Provincial People's Hospital, Chengdu, China.

**Keywords:** conservative interventions, network meta-analysis, postpartum urinary incontinence, study protocol

## Abstract

**Background:**

Postpartum urinary incontinence (PPUI) is a common urological condition in women after childbirth. Due to the side effects of surgical and pharmacological therapies, the patients and physicians alike express a strong preference for conservative approaches on PPUI, such as pelvic floor muscle training, biofeedback, electrical stimulation, bladder training, vaginal cones and acupuncture. Application of these conservative approaches should be guided by high quality evidence, yet their comparative effectiveness has not been well documented. Therefore, the network meta-analysis aims to compare, rank and summarize all available studies to determine which conservative intervention is more effective for PPUI.

**Methods:**

In this present study, qualified English and Chinese studies will be searched in PubMed, Scopus, EMBASE, The Cochrane Library, Web of Science, VIP Database, Wanfang Database, Chinese Biomedical Literature Database and China National Knowledge Infrastructure. All eligible randomized controlled trails (RCTs) of conservative interventions for PPUI will be included. R software 3.61 (R Foundation for Statistical Computing, Vienna, Austria) will be applied to synthesize data and conduct network meta-analysis. *I*^2^ statistic and *Z* test will be used to assess heterogeneity and inconsistency, respectively.

**Results:**

Ethical approval is not required for this existed literature based meta-analysis. The findings of this research will be disseminated through a recognized journal.

**Conclusion:**

The findings of this study will provide ranking evidence for clinicians and patients to choose a more appropriate conservative therapy on PPUI.

**Trial registration number:**

PROSPERO CRD42020168042

## 1 Introduction

Postpartum urinary incontinence (PPUI) is a frequent urological condition in females after childbirth,^[1]^ with negative influence on social, physical and mental lives of women,^[2]^ and also, which is costly from personal and social economic perspectives.^[3]^ The significant symptom of PPUI is involuntary urinary leakage, especially during sneezing, coughing or exercise.^[4]^ Delivery and neonatal parameters such as vaginal delivery, prolonged labor, parity and high birthweight, as well as maternal ageing and obesity are main risk factors involved in the occurrence of PPUI.^[5–7]^ Epidemiological studies have showed that the prevalence rates of PPUI vary from 3% to 34%,^[8,9]^ nearly 73% of women suffered from PPUI were still reported to have persistent urinary incontinence symptom even 6 years after giving birth.^[10]^ In previous survey in middle-aged and old women in China, we even revealed that 40% women with PPUI would suffer from urinary incontinence in the future.^[11]^ Obviously, PPUI has been raised as a significant public health problem, and therefore, it is reasonable for us to validate an effective approach for PPUI, to improve the life quality of patients.

Treatment methods of PPUI include surgical interventions, pharmacological interventions and conservative interventions. Although surgical interventions are demonstrated to be effective, complications related to the utilization of synthetic materials have limited the application of surgery.^[12]^ Moreover, the American Food and Drug Administration has released the notification of warning the use of these surgical devices for urinary incontinence on July 2011, which further contributed to the declination of the proportion of patients who received surgical method for PPUI.^[13]^ Pharmaceutical approaches, such as alpha-adrenergic agonist, hormone replacement treatment, are available drug therapies for urinary incontinence.^[14]^ However, the insufficient efficacy and significant side effects limit the wide use of these pharmaceutical approaches. Therefore, conservative interventions have become the first-line option for managing women with PPUI.

Multiple conservative methods for PPUI are available, such as pelvic floor muscle training (PFMT), biofeedback (BF), electrical stimulation (ES), bladder training (BT), vaginal cones (VC) and acupuncture. Various researches have showed that PFMT could increase urethral closure pressure and stabilize the bladder neck by squeezing the related muscles, consequently, the instances of urinary leakage were mitigated. Hence, PFMT is recommended as a non-invasive and easily administered method for PPUI.^[1,15,16]^ BF is a technique usually combined used with PFMT, which help the patient learn to control the pelvic floor muscle function.^[17]^ In short-time postpartum period, BF plus PFMT therapy could effectively improve pelvic floor muscle strength and manage symptom of urinary incontinence.^[18]^ Some researchers have utilized ES to stimulate the pelvic floor muscles and nerves by transmitting different current intensities, to improve the ability of controlling urine in the pelvic floor rehabilitation programme for primiparous women.^[19]^ BT focuses on increasing the bladder capacity by lengthening the interval time between bathroom trips, to improve the detrusor stability during urine storage period.^[20]^ A RCT trial emphasized the value of BT for controlling urinary incontinence in postpartum period either alone or with PFMT.^[21]^ VC therapy provides an alternative easy way for women who are not able to adequately contract their pelvic floors when performing PFMT.^[22]^ A quantitative systematic review has conducted to evaluate the performance of VC for treating urinary incontinence in postpartum women, the result of which has demonstrated the efficacy of VC for strengthening pelvic floor muscle.^[23]^ Acupuncture has been performed in eastern Asia for thousands of years and is still broadly practiced around the world today. Considerable studies have been conducted to demonstrate that acupuncture exhibit favorable effects on urinary incontinence by mitigating bladder instability.^[24]^

It is clearly seen that strong evidence was needed to back the optimal decision which was made from all these conservative interventions in clinical practice. However, the relative efficacy between all these approaches has not been well established until now. Therefore, a network meta-analysis is urgently needed to fill the vacuum of scientific uncertainty existed in the field of PPUI treatment. By using the systematic review and network meta-analysis, we can compare and rank the indirect results of conservative interventions for treating PPUI in different studies. Moreover, we also aim to provide a precise and systematic conclusion and interpretation based on the current researches through this study.

## 2 Methods

### 2.1 Design and registration

The present systematic review and network meta-analysis, aiming to assess the comparative effectiveness and safety of conservative therapies for PPD, is designed under the guideline of Preferred Reporting Items for Systematic Review and Meta-Analysis Protocols^[25]^ and registered on PROSPERO (registration number CRD42020168042). The findings of this systematic review and network meta-analysis are expected to be published in a recognized journal in accordance with the guideline of Preferred Reporting Items for Systematic Reviews and Network Meta-Analysis.^[26]^

### 2.2 Ethics

Formal ethical approval is not needed, because the present study is a meta-analysis based on existed studies.

### 2.3 Eligibility criteria

#### 2.3.1 Type of studies

All RCTs of conservative interventions for PPUI will be considered for inclusion, regardless of blinding. The clinical trials without a comparative group will not be included. We only include the studies written in English or Chinese.

#### 2.3.2 Type of participants

Participants were primiparous or multiparous postnatal women with a clinically confirmed diagnosis of PPUI, regardless of age or race.

#### 2.3.3 Type of interventions and comparators

Experimental interventions: Any types of conservative therapies in treating PPUI will be considered for inclusion, such as PFMT, BF, ES, BT, VC and acupuncture and so on. There is no restriction in duration of treatments. Studies with concomitant use of different conservative treatments will be included, while researches combining conservative interventions and pharmacological or surgical interventions will not be included in our study. Control interventions: Placebo, waiting-list control or non-treatment control in comparative group will be included in our study, being regarded as a single node in network meta-analysis. Studies with any of the described conservative interventions in control group will also be included.

#### 2.3.4 Type of outcome measures

Primary outcomes: The main outcome is the symptom improvement of the amount of urine leakage measured by the 1-hour pad test from baseline to endpoint. Additional outcomes: The improvement of incontinence-specific quality of life and clinician's observations will be set as our secondary outcomes. The incontinence-specific quality of life was evaluated by Incontinence Quality of Life Questionnaire, or any other valid scoring system. The clinician's observations include observation of urinary incontinence; and urodynamic examination. In addition, side effects will be considered as additional outcome.

#### 2.3.5 Exclusion criteria

Duplicate literature and studies with unavailable data will be excluded in our research. We will also exclude the RCTs with cross-over design or quasi-random allocation.

### 2.4 Search strategy

We will conduct a comprehensive search in EMBASE, PubMed, Scopus, Web of Science and The Cochrane Library to identify available English data. Chinese electronic bibliographic database resources will also be searched for Chinese data, including the VIP database, Wanfang Database, Chinese Biomedical Literature Database, and China National Knowledge Infrastructure. In addition, reference lists of eligible studies and international trial registry websites will also be searched as supplementary gray literature.

We have provided a preliminary search strategy of PubMed in Table 1, which will also be used in other electronic databases after adjustment.

**Table 1:**
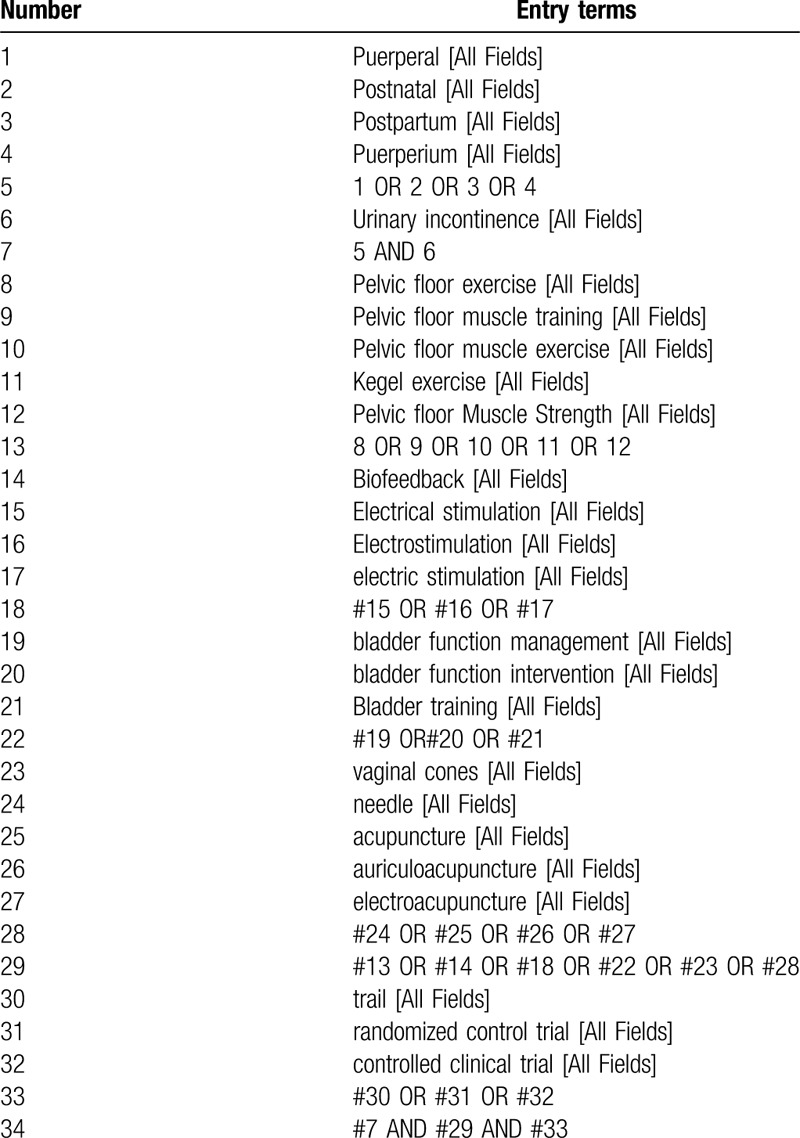
Search strategy applied in PubMed.

### 2.5 Study selection and data extraction

#### 2.5.1 Study selection

**Figure 1:**
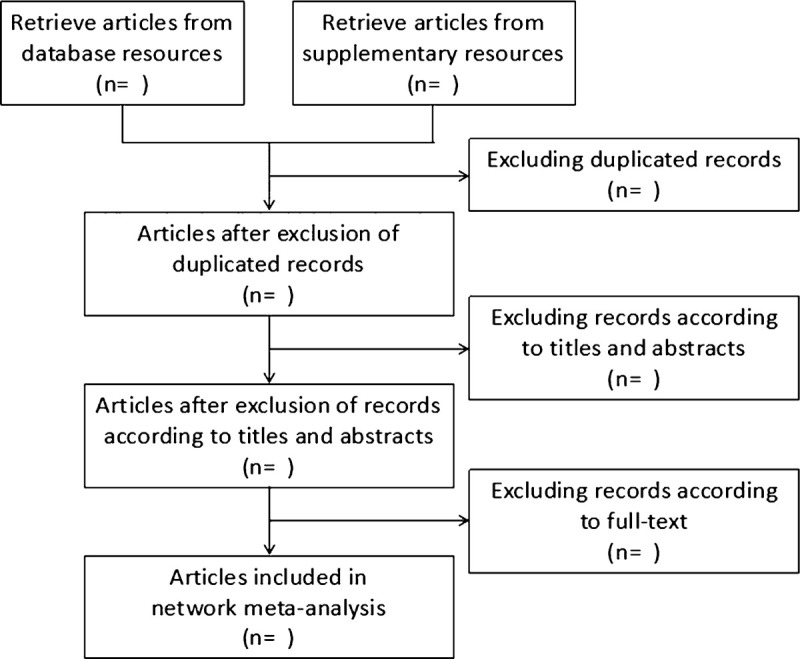
Flow chart of network meta-analysis for conservative interventions for postpartum urinary incontinence.

All studies retrieved according to the pre-designed search strategy will be imported into Endnote X7. We will remove the duplicate articles firstly. Then the titles and abstracts of studies will be reviewed to select available RCTs in accordance to the pre-defined eligibility criteria. Afterward, further assessment will be conducted by reading full-text. All these procedures will be performed by 2 independent reviewers and documented in flow chart (Fig. 1) under the guideline of Preferred Reporting Items for Systematic Reviews and Network Meta-Analysis. A third reviewer will be consulted with in case of disagreements.

#### 2.5.2 Data extraction

Using 3 initial studies to pilot and refine, 2 reviewers will establish standard extraction sheet by Microsoft Excel 2013 to extract data. The study information (eg, first author, title, journal, country, year published, and methods of randomization and blinding), participant information (eg, age, race, gender, and number), intervention information (eg, type of treatment, frequency, duration of therapy, and comparative group), and outcomes (including primary and secondary outcomes) will be extracted by the 2 reviewers independently.

If there are any disagreements, a third reviewer will be invited to consult with. In case of any missing data, we will try to get touch with the authors by email or telephone to gain the full data.

### 2.6 Assessment of risk of bias

Two authors will evaluate the methodological quality of selected studies independently in accordance to the Cochrane Handbook, which comprises of 7 domains (random sequence generation, allocation concealment, blinding of participants, personnel and outcome assessors, incomplete outcome data, selective reporting, and other sources of bias). Each domain will be classified as high, unclear or low risk of bias. If there are any variations in opinion, a third reviewer will be consulted with to address the disagreements.

### 2.7 Data synthesis and statistical methods

Before data synthesis, *I*^2^ statistic will be applied to analyze the homogeneity, checking whether the included articles are mergeable. If *I*^2^ ≤ 50%, the homogeneity is acceptable, and the fixed effect model will be used to combine data. Conversely, the data will be combined by random effect model.

The effect size of continuous variable data will be presented as standardized mean difference and related 95% confidence intervals, while effect size of categorical variable data will be expressed as risk ratio and related 95% confidence intervals. For direct comparisons, conventional pairwise meta-analysis will be performed, while network meta-analysis will be conducted for indirect comparisons. R software 3.6.1 (R Foundation for Statistical Computing, Vienna, Austria) and related “netmeta” package will be applied in our analysis. *Z* test will be used to evaluate the inconsistency between direct and indirect comparative results. The findings of direct and indirect comparisons will be presented by drawing a network diagram.

The potential subgroup analysis should be performed in case of high heterogeneity across trails. Sensitivity analysis will also be considered to verify the robustness of the findings. Additionally, funnel plots and Egger Regression test will also be performed to evaluate the potential publication bias.^[27]^

### 2.8 Grading the quality of evidence

In accordance to the standards of Grading of Recommendations Assessment, Development and Evaluation, 2 authors will grade the evidence quality as high, medium, low, and very low.^[28]^

## 3 Discussion

With a considerable amount of publications on conservative interventions for patients with PPUI, we want to find out which treatment was the relatively better 1 among all these interventions. Given that network meta-analysis can offer the evidence based high quality results by comparing, ranking and summarizing different indirect comparisons, we attempt to use network meta-analysis to select a more appropriate conservative treatment for PPUI. Therefore, by referring to the previous work,^[29]^ we conceived and designed this network meta-analysis protocol. We also hope our result will provide a wider insight into the field of PPUI treatment, which, to some extent, can provide a reference for clinicians and patients.

## Author contributions

**Conceptualization:** Tianmin Zhu, Yang Wang, Yunlu Liu.

**Data curation:** Yalin Chen, Mimi Qiu.

**Formal analysis:** Yang Wang, Hui Li.

**Funding acquisition:** Tianmin Zhu, Yunlu Liu.

**Investigation:** Yang Wang, Hui Li, Yalin Chen, Mimi Qiu, Jun Wang, Qinghong Hao, Yang Tu.

**Methodology:** Yang Wang, Wei Peng.

**Software:** Yang Wang, Wei Peng.

**Supervision:** Hui Li, Tianmin Zhu.

**Writing – original draft:** Yang Wang.

**Writing – review & editing:** Yunlu Liu, Tianmin Zhu.
